# Burden and predictors of hypertension in India: results of SEEK (Screening and Early Evaluation of Kidney Disease) study

**DOI:** 10.1186/1471-2369-15-42

**Published:** 2014-03-06

**Authors:** Youssef MK Farag, Bharati V Mittal, Sai Ram Keithi-Reddy, Vidya N Acharya, Alan F Almeida, Anil C, Ballal HS, Gaccione P, Issacs R, Sanjiv Jasuja, Kirpalani AL, Kher V, Modi GK, Georgy Nainan, Jai Prakash, Mohan M Rajapurkar, Rana DS, Rajanna Sreedhara, Sinha DK, Bharat V Shah, Sham Sunder, Raj Kumar Sharma, Sridevi Seetharam, Tatapudi Ravi Raju, Ajay K Singh

**Affiliations:** 1 Renal Division, Brigham & Women’s Hospital & Harvard Medical School, 75 Francis Street, Boston, MA 02115, USA; 2 NKF-India, Mumbai, India; 3 Hinduja Hospital, Mumbai, India; 4 Vivekananda Memorial Hospital, H.D.Kote, Saragur, Mysore, India; 5 Manipal Institute of Nephrology & Urology, Bangalore, India; 6 Deep Hospital, Ludhiana, India; 7 Indraprastha Apollo Hospital, New Delhi, India; 8 Bombay Hospital, Mumbai, India; 9 Fortis Flt. Lt. Rajan Dhall Hospital, New Delhi, India; 10 Bhopal Memorial Hospital & Research Center, Bhopal, India; 11 PVS Memorial Hospital, Cochin, India; 12 Institute of Medical Sciences, BHU, Varanasi, India; 13 Muljibhai Patel Urological Hospital, Nadiad, India; 14 Sir Ganga Ram Hospital, New Delhi, INdia; 15 Wockhardt Hospitals, Bangalore, India; 16 Kanpur Rotary Kidney Foundation, Kanpur, India; 17 Lilavati Hospital, Mumbai, India; 18 Dr. R.M.Lohia Hospital, New Delhi, India; 19 Sanjay Gandhi Postgraduate Institute of Medical Sciences, Lucknow, India; 20 Andhra Medical College & King George Hospital, Vishakhapatanam, India

**Keywords:** Hypertension, CKD, Awareness

## Abstract

**Background:**

Hypertension (HTN) is one of the major causes of cardiovascular morbidity and mortality. The objective of the study was to investigate the burden and predictors of HTN in India.

**Methods:**

6120 subjects participated in the Screening and Early Evaluation of Kidney disease (SEEK), a community-based screening program in 53 camps in 13 representative geographic locations in India. Of these, 5929 had recorded blood pressure (BP) measurements. Potential predictors of HTN were collected using a structured questionnaire for SEEK study.

**Results:**

HTN was observed in 43.5% of our cohort. After adjusting for center variation (p < 0.0001), predictors of a higher prevalence of HTN were older age ≥40 years (p < 0.0001), BMI of ≥ 23 Kg/M^2^ (p < 0.0004), larger waist circumference (p < 0.0001), working in sedentary occupation (p < 0.0001), having diabetes mellitus (p < 0.0001), having proteinuria (p < 0.0016), and increased serum creatinine (p < 0.0001). High school/some college education (p = 0.0016), versus less than 9th grade education, was related with lower prevalence of HTN. Of note, proteinuria and CKD were observed in 19% and 23.5% of HTN subjects. About half (54%) of the hypertensive subjects were aware of their hypertension status.

**Conclusions:**

HTN was common in this cohort from India. Older age, BMI ≥ 23 Kg/M^2^, waist circumference, sedentary occupation, education less, diabetes mellitus, presence of proteinuria, and raised serum creatinine were significant predictors of hypertension. Our data suggest that HTN is a major public health problem in India with low awareness, and requires aggressive community-based screening and education to improve health.

## Background

The burden of hypertension varies remarkably throughout the regions of the world and is a serious public health problem in both developed and developing countries [1,2]. Both systolic and diastolic hypertension are important predicting risk factors of cardiovascular disease, chronic kidney disease and stroke. [3] World Health Organization (WHO) data indicate that by 2025 the global burden of hypertension will increase by 60% to be 1.56 billion individuals worldwide and higher in the developed nations [4]. Lopez et al. have shown that 5.3 million deaths were attributable to cardiovascular disease in the western world as compared to 8 to 9 million in the developing world [5]. According to a recent report, hypertension was the third major cause of disease burden, in both developed and developing regions worldwide, with 64 million disability adjusted life years (DALY) [6].

Hypertension is one of the most important modifiable risk factors for cardiovascular disease (CVD) [7]. Hypertension in early stages does not show any symptoms; hence many are unaware of its presence. The prevalence of hypertension is increasing and it correlates with the chronic kidney disease in the US [9,10]. Early detection is feasible using a simple and accurate screening test and aggressive blood pressure management. Yet this has not received adequate attention or allocation of public health resources for planning effective preventive strategies. In India, as a developing country with a population estimated at 1.1 billion, the prevalence of hypertension has been estimated to be 3% to 34.5% in males and 5.8% to 33.5% of females [10-13].

Understanding the burden of hypertension, as a preventable disease, and underlying risk factors by organizing population screening is the first step towards planning any effective preventive programs. The Screening and Early Evaluation of Kidney Disease (SEEK Study) aimed at generating epidemiological information on chronic kidney disease and its associated non-communicable disease in India. This article presents the results from SEEK Study on the prevalence and risk factors of hypertension.

## Methods

### Assembly of the SEEK cohort

Thirteen academic and private medical centers in India participated in the study under the name of “Screening and Early Evaluation of Kidney disease- SEEK”. It was conducted between June 2005 to May 2007, coordinated from the Brigham and Women’s Hospital in Boston, Massachusetts. The protocol was approved by the Partner’s Institutional Review Board (IRB) as well as by individual centers’ own institutional IRBs in India. Signed or verbal informed consent (confirmed by a witness) was obtained before administering the questionnaire, taking measurements or blood collection. The database is based at the Brigham and Women’s Hospital.

Geographic distribution of the centers is shown in Table 1. In general, sampling of subjects from all parts of India except the Eastern region was achieved. The camps were conducted in a community-based setting, and any Indian above the age of 18 years was eligible to participate in the screening. Participants voluntarily walked in the screening camp as they noticed the flyer. Six of the 13 centers screened an exclusive urban population, 5 screened an exclusively rural population; and two screened a mixture. The urban population in our study comprised 51.2% and 48.8% were rural. Of the 6120 screened subjects, 6047 (98.8%) were adults and without any prior history of renal replacement therapy or dialysis. Of these 5929 subjects (98%) had blood pressure measurements, comprising the analysis population for this article.

**Table 1:** Centers for the SEEK study

**Center**	**Screened population**
**North zone**	
Himachal Pradesh*	520
Varanasi, Uttar Pradesh*	515
Kanpur, Uttar Pradesh§	511
Apollo Hospital, Delhi§	472
RML Hospital, Delhi§	280
Ludhiana, Punjab§	402
**West-Central zone**	
Nadiad, Gujarat*	506
Bombay, Maharashtra§	531
Bhopal, Madhya Pradesh✞✞	438
**South zone**	
Cochin, Kerala§	497
H.D.Kote, Karnatak*	1022
Bangalore, Karnatak✞	275
Vizag, AndhraPradesh*	152
Total	6120

*Centers that screened 100% rural subjects.

§Centers that screened 100% urban subjects.

✞Center screened 73% urban, 27% rural subjects.

✞✞Center that screened 50% each of urban and rural population.

### Data collection and participant selection

A structured questionnaire translated into local languages was used (Additional file 1). A team of nephrologists, nurses, technicians and interviewers participated in the camps. At every site, staff was trained in interview techniques and measurement of height, weight and blood pressure by organizing a half day workshop prior to the camp. Body mass index (BMI) was calculated using the formula “weight (Kg)/height (M^2^).” Overweight was defined as BMI ≥ 23 and < 27.5 Kg/M^2^, obese as BMI ≥ 27.5 Kg/M^2^[12]. We also calculated the prevalence of being overweight and obese using the conventional cut off of BMI ≥25 Kg/M^2^ and ≥30 Kg/M^2^ respectively. The waist to hip circumference ratio (WHR) was calculated by using the waist circumference at the narrowest circumference between the lower costal margin and the iliac crest. Hip circumference was measured at the maximum circumference at the level of the femoral trochanters. Abdominal obesity was defined as ≥ 90 cms for males and ≥ 80 cms for females (Asian cut off) [14].

### Blood Pressure (BP) measurements

In order to get a standardized BP measurement, a protocol per American Heart Association guidelines [15] and a power-point presentation was provided to the centers, and staff training on measuring the blood pressure was carried out prior to camps. Systolic blood pressure (SBP) was based on the 1st Korotkoff phase and diastolic (DBP) on the 5th Korotkoff phase. Mercury sphygmomanometer was used after checking for zero error. After rest for 5 minutes, BP was recorded in the sitting position in the right arm supported at heart level, to the nearest 2 mm using mercury sphygmomanometer. An average of two readings was taken into consideration.

### Laboratory work-up

Non-fasting blood samples were collected in the camps. Blood was sent to a central laboratory. Quality control for temperature transporting specimens was checked and confirmed; i.e., 4–9 degree Celsius. Serum creatinine was measured using Jaffe Colorimetric method on a Roche Hitachi 912 analyzer. The instrument was calibrated (external calibration) using the Cleveland Clinic Foundation (CCF) creatinine panel. Regression analysis was carried out to calculate a formula to convert creatinine values obtained at the SRL-Ranbaxy laboratory (SRL) to the CCF values as follows: CCF creatinine = -0.13 + SRL creatinine * 0.99. Urine protein was detected by dipstick method (Bayer Multistix 10 SG). A modified MDRD-3 equation GFR (mL/min/1.73 m^2^) = 175 × (S_cr_)^-1.154^ × (Age)^-0.203^ × (0.742 if female) × (1.212 if African American) was used [16]. Plasma glucose was measured by the glucose oxidase peroxidase method using Roche Hitachi 912 analyzer.

### Definition of variables

Urine protein positivity (proteinuria) was defined as urine protein 1+ or more. Elevated blood pressure (EBP) was defined as SBP/DBP ≥ 140/90 mmHg [8]. Hypertension was defined as SBP/DBP ≥ 140/90 mmHg [8] or if the patient was on medication for hypertension or had a positive self reported history of hypertension (based on a response to “have you ever been told that you have high blood pressure” or ‘a past history of high blood pressure”). Prehypertension was defined as a systolic pressure from 120 to 139 millimeters of mercury (mmHg) or a diastolic pressure from 80 to 89 mmHg [8]. Diabetes was defined as fasting blood sugar FBS > 126 or non-fasting blood sugar ≥ 200 or on any medications for diabetes mellitus (ADA definition) [17], or if there was a positive response to the questions “have you ever been told that you have diabetes” or “past history of diabetes”. Self reported history of medications was verified and if the subject did not know the name of the medication and/or if the stated name was incorrect, the response was considered as “no” even if the subject’s response to the question “are you on BP or diabetes medications” was “yes”. CKD stages were defined using NKF-KDOQI guidelines (eGFR < 60 ml/min/1.73 m^2^ or proteinuria ≥ 1+ on dipstick) [18]. Self reported ischemic heart disease was taken as present if there was a self reported history of a myocardial infarction, percutaneous angioplasty or coronary artery bypass surgery. Family history included only first-degree relatives.

### Statistical analysis

SAS Statistical Analysis Software Version 9.1.3 (SAS Institute Inc., Cary, NC, USA) was used. Univariate analyses comparing distributions of socio-demographic and clinical/historical measures between HTN groups was performed first, with comparisons made using Fisher’s Exact Test and t-tests. Then a multivariate logistic regression model, with HTN yes or no as the outcome, was fit with variables correlating highly with it univariately (p < 0.05). All were entered into the model, and a stepwise selection process combined with finding a parsimonious minimum of the Akaike and Schwarz model information criteria [19] resulted in the final composition of the model. All covariates are adjusted simultaneously for the others including one other covariate, family history of HTN, which was forced to remain in the model. Due to the number of variables and the inclusion of the center variable interactions were not tested. We also presented the adjusted odds ratios and the corresponding 95% confidence intervals for these resulting relationships.

## Results

The main finding of this study is that hypertension prevalence in our SEEK cohort is 43.5%, and 41.5% of subjects had blood pressure in the range of “pre-hypertension”. The mean SBP and DBP of the subjects was 126.8(20) and 80.4(11) mmHg respectively (Table 2).

**Table 2:** Demographic features

**Characteristics**		**HTN-Yes**	**HTN-No**	**p value**
		**(43.5%)**		
Age	n	2578	3351	**<.0001**
	Mean (SD)	52.1(14.02)	40.0(13.93)	
BMI	n	2561	3340	**<.0001**
	Mean (SD)	25.7(5.37)	22.7(4.87)	
Waist circum.	n	2426	3236	**<.0001**
	Mean (SD)	88.3(13.33)	79.3(13.12)	
Hip circum	n	2425	3234	**<.0001**
	Mean (SD)	96.1(12.21)	89.8(11.07)	
Systolic BP	n	2577	3351	**<.0001**
	Mean (SD)	140.7(19.97)	116(10.99)	
Diastolic BP	n	2575	3344	**<.0001**
	Mean (SD)	87.7(10.97)	74.8(7.58)	
S.Creatinine	n	2453	3220	**<.0001**
	Mean (SD)	1.1(1.10)	0.9(0.34)	

Boldfaced p values means “statistically significant”.

**Figure 1:**
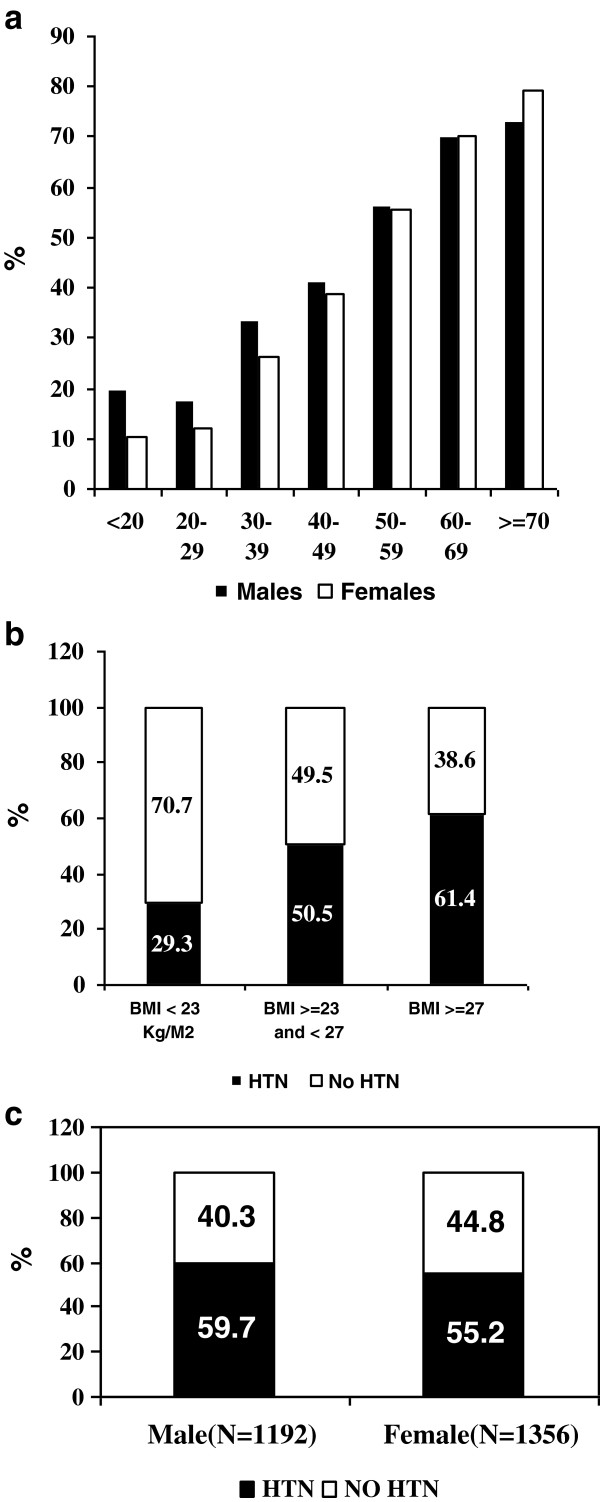
**Prevalence of Hypertension by Subgroups. a)** Prevalence of HTN by age and gender. **b)** Prevalence of HTN among lean/ normal, overweight and obese subjects as defined by Asian cutoff. **c)** Risk of HTN among males and females with abdominal obesity as defined by Asian cutoff.

The demographic and clinical history characteristics of the screened population are shown in Tables 2 and 3. The mean (SD) age of the screened population was 45.3(15.2) years and 55% were males. Increasing prevalence with age was observed, with 70% of males and females 60 to 70 years of age having hypertension. In the > 70 years age group there was a higher percentage of females having HTN. (Figure 1a) Mean BMI, waist and hip circumference were 24(5.3) Kg/M^2^, 83.2(13.9) cm, and 92.5(12) cm respectively. Forty three percent showed indications of abdominal obesity. Forty three percent of the subjects had < 8th grade education, 32.1% had “a 9th grade to some college” education, and 24.3% had graduate or postgraduate educations. About one fifth (21.8%) were involved in manual labor (agricultural workers, laborers, and cowherds), 33.6% had salaried jobs/ their own business, 2.4% were students and 41.3% were involved with sedentary occupations (homemakers, unemployed, retired or other occupations), with 63.6% earning less than $125 per month. Another fifth (21.6%) were current smokers or tobacco chewers; only 2.3% did both. Seventeen percent of subjects had diabetes (14% self-reported), 21.2% reported having a family history of HTN, 13.5% had proteinuria, and 16.3% had CKD. Self reported ischemic heart disease (heart attack, angioplasty or bypass surgery) was observed in 5.4% of the subjects, self-reported stroke in 1.3% and self-reported peripheral vascular disease in 2.4%. Serum creatinine levels averaged 1.0 mg/dL.

**Table 3:** Prevalence of risk factors in HTN population

**Characteristic**	**Result**	**HTN-Yes**		**HTN-No**		**p-value**
		**N = 2578**		**N = 3351**		
		**n**	**%**	**n**	**%**	
**Age**	< 40 years	515	20	1701	50.8	
	≥ 40 years	2063	80	1650	49.2	**<0.0001**
**Gender**	Female	1095	42.5	1572	46.9	
	Male	1482	57.5	1779	53.1	**0.0007**
**Education**	≤ 8th grade	1011	39.2	1540	46.0	
	9th to some college	837	32.5	1069	31.9	
	College grad/Post grad	715	27.7	724	21.6	**0.0007**
**Occupation**	Manual labor*	391	15.2	899	26.8	
	Own business/salaried	867	33.6	1128	33.7	
	Student	18	0.7	122	3.6	
	Sedentary**	1275	49.5	1171	34.9	**0.0007**
**Income**	≤ 125 $/month	1477	57.3	2295	68.5	
	> 125 $/month	980	38.0	939	28.0	**<0.0001**
**Urabn**		1502	58.3	1535	45.8	
**Rural**		1076	41.7	1815	54.2	**<0.0001**
**Obesity status:**						
**Body mass index**✝	< 23 Kg/M ^2^	789	30.6	1904	56.8	
	≥ 23 Kg/M ^2^	1772	68.7	1436	42.9	**<0.0001**
**Body mass index**✝✝	< 27 Kg/M ^2^	1709	66.3	2804	83.7	
	≥ 27 Kg/M ^2^	852	33.0	536	16.0	**<0.0001**
**Body mass index**✝✝✝	< 30 Kg/M ^2^	2100	81.5	3090	92.2	
	≥ 30 Kg/M ^2^	461	17.9	250	7.5	**<0.0001**
**Abdominal**	No	965	37.4	2149	64.1	
**Obesity**⊙	Yes	1461	56.7	1087	32.4	**<0.0001**
**Current Smoker or tobacco chewer**	No	1875	72.7	2418	72.2	
	Yes	516	20.0	765	22.8	**0.0312**
**Current Smoker & tobacco chewer**	No	2322	90.1	3054	91.1	
	Yes	45	1.7	94	2.8	**0.0117**
**Self reported DM**	No	1892	73.4	2905	86.7	
	Yes	570	22.1	263	7.8	**<0.0001**
**Diabetes mellitus♦**	No	1801	69.9	2862	85.4	
	Yes	672	26.1	343	10.2	**<0.0001**
**Family H/o HTN**	No	1967	76.3	2697	80.5	
	Yes	607	23.5	647	19.3	**<0.0001**
**Urine-protein**	Absent/trace	2045	79.3	2981	89.0	
	> = 1+	489	19.0	310	9.3	**<0.0001**
**CKD♦ ♦**	No	1801	69.9	2814	84.0	
	Yes	606	23.5	358	10.7	**<0.0001**
**Center type**	Urban	1355	52.6	1284	38.3	
	Rural	974	37.8	1618	48.3	
	Mixed population	249	9.7	449	13.4	**0.0051**

P value < 0.05 is significant.

* - includes categories of occupation – agriculture workers, laborers, cowherds.

** - includes, homemakers (housewives), retired, unemployed and others.

✝ - Body Mass Index - ≥ 23 Kg/M^2^ includes overweight and obese as per Asian cutoff.

✝✝ - Obese as per Asian cutoff.

✝✝✝ - Obese as per conventional cutoff.

⊙ - Waist circumference ≥ 90 cms in males or ≥ 80 cms in females.

**♦** - Diabetes Mellitus (DM) = Fasting blood sugar ≥ 126 or non-fasting blood sugar ≥ 200 or if self reported D.M or on anti diabetic medication.

**♦ ♦ -** MDRD-GFR < 60 ml/min/1.73 m^2^ or proteinuria ≥ 1+ on dipstick.

**Figure 2:**
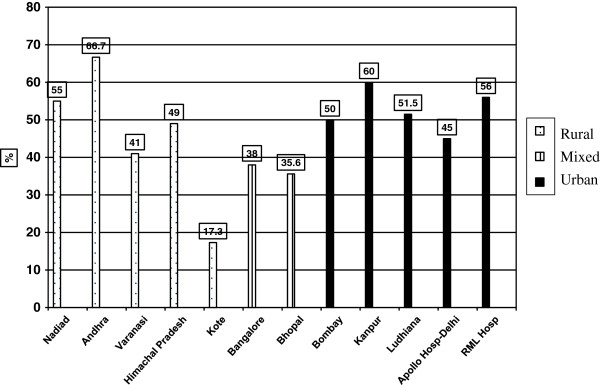
Prevalence of hypertension by center.

The prevalence of hypertension at various centers is depicted in Figure 2. Among the rural centers, Kote had the lowest prevalence of HTN, with the other rural centers having rates comparable to those in some of the urban centers. Given that Kote screened 1/3 of the total rural population, the result of lower rural prevalence compared to urban prevalence is probably due to Kote, and highlights the need for center as an adjuster in the multivariate model.

Only 54% of the HTN subjects were aware of their hypertension and reported positively to the question “Have you ever been told that you have high blood pressure?” or “Do you have a past history of hypertension”. 21.7% of the hypertensive population was on treatment.

### Comparisons of HTN groups

The mean age of hypertensive subjects was 52.1 years (SD 14.02 years), significantly higher than the non-hypertensive subjects (p = <0.0001). Eighty percent of subjects with HTN were ≥40 years of age as compared to 49.2% of those without HTN (p < 0.0001). Figure 1a shows increasing prevalence of HTN with age; in the 6^th^and 7^th^ decade ≥70% of males and females in the screened population had hypertension. The mean systolic and diastolic blood pressure of the hypertensive group were 141(20) and 88(11) mmHg respectively, while that of the non hypertensive group was 116(11) and 74(8) mmHg respectively (p < 0.0001). Approximately fifty seven percent of subjects with hypertension were males and to 42.5% were females (p = 0.0007). For the HTN group, the mean BMI was 25.7(5.4) Kg/M^2^, the mean waist circumference was 88.3(13.3) cm, the hip circumference 96.1(12.2) cm, and 56.7% had abdominal obesity; these numbers were significantly higher than in the non-hypertensive group (p < 0.0001 for all 4 parameters). There was a significant difference in the distribution of educational, occupational, and income levels between the groups, as well as who smoked and/or chewed tobacco. Family history of HTN was reported more often in the HTN group (23.5% vs. 19.3%, p < 0.0001).

Confirmed and self-reported diabetes (26.1% and 22.1%, respectively) were both higher in the HTN group (p < 0.0001). proteinuria was observed among 19% of HTN subjects as compared to 9.3% of non hypertensive subjects (p < 0.0001). ). CKD (MDRD GFR < 60 ml/min/1.73 m^2^ or GFR ≥ 60 ml/min/1.73 m^2^ and urine protein ≥ 1+) was found in 23.5% of HTN subjects and 10.7% of non-hypertensive subjects (p < 0.0001).

A significantly higher prevalence of cardiovascular complications was observed among subjects with HTN as compared with those without (see Table 4). Self reported ischemic heart disease (heart attack, angioplasty or bypass surgery) was observed in 8.9% (p < 0.0001), stroke in 1.9% (p = 0.0002) and peripheral vascular disease in 3.4% (p = 0.005) of HTN subjects, respectively.

**Table 4:** Cardiovascular Disease in the SEEK Cohort (self reported history)

**Characteristics**	**HTN Yes**		**HTN No**		**Total population**		**p-value**
	**N = 2578**		**N = 3351**		**N = 5929**		
	**n**	**%**	**n**	**%**	**n**	**%**	
Ischemic heart disease*	229	8.9	90	2.7	319	5.4	<0.0001
**Stroke**	50	1.9	27	0.8	77	1.3	0.0002
**Peripheral vascular disease**	87	3.4	57	1.7	144	2.4	0.0051

*Self reported positive history of heart attack, angioplasty or coronary by-pass operation.

Hypertension was significantly higher in the urban population compared to the rural population (p < 0.0001), and the center distribution showed statistically significant differences among the three center types (urban, rural, mixed) (p = 0.005).

### Predictors of hypertension

We combined the variables described above into a multivariate model, and this analysis confirmed the indications of the independent tests. Those greater than 40 years of age were 2.24 times more likely to have HTN and males 1.17 times more likely to have HTN than females. This cutoff was chosen after repeated univariate modeling at each year of age showed 40 to depict the largest contrast in HTN prevalence. Continuous measures of waist circumference retained a positive association with HTN (p < 0.0001). Having a 9th grade +/some college education resulted in a lower chance of being hypertensive (OR: 0.76, 95%CI: 0.64- 0.90, p = 0.0016) compared to having a less than 9th grade education. Those involved in sedentary (p < 0.0001) occupations versus an agricultural occupation were more likely to have HTN. Individuals with diabetes were 1.71 times more likely to become hypertensive, those with proteinuria 1.40 times more likely, and higher serum creatinine predisposed one to being hypertensive. Family history of hypertension was not significantly related to HTN multivariately, but was left in the final model (p = 0.20). Table 5 has a complete list of odds ratios and p-values. The strongest predictors of hypertension were age more than 40 years, sedentary life style, diabetes, high serum creatinine, and the presence of urine protein. There was significant center to center variation in the prevalence of hypertension when the 12 centers were compared with the center Bombay (an urban center with 50% prevalence of hypertension, (p <0.0001).

**Table 5:** Multi-variate regression analysis for association of risk factors with HTN (adjusted for all characteristics with p value < 0.05 on univariate analysis)

**Variable**	**Adjusted odds ratio**	**p-value**
> = 40 years	2.24 (1.93- 2.59)	<0.0001
*(ref: less than 40 years)*		
Male	1.17 (1.00- 1.38)	0.0564
*(ref: female)*		
9th Grade/Some College	0.76 (0.64- 0.90)	0.0016
*(ref: less than 9th grade)*		
College Grad/Post Grad	0.85 (0.70- 1.04)	0.1159
*(ref: less than 9th grade)*		
Own Business/Salaried	1.07 (0.87- 1.32)	0.5149
*(ref: unemployed)*		
Student	0.80 (0.43- 1.48)	0.4751
*(ref: unemployed)*		
Sedentary*	1.79 (1.45- 2.20)	<0.0001
*(ref: physical job)*		
BMI > =23 kg/m^2	1.37 (1.15- 1.64)	0.0004
*(ref: BMI less than 23)*		
Waist Circumference	1.03 (1.03- 1.04)	<0.0001
*(ref: normal WC)*		
Diabetes Mellitus**♦**	1.71 (1.44- 2.04)	<0.0001
*(ref: no DM)*		
Family History of HTN	1.11 (0.95-1.31)	0.1963
*(ref: No FH of HTN)*		
Serum Creatinine	1.61 (1.34- 1.93)	<0.0001
*(ref: normal creatinine)*		
Urine protein > = 1+	1.40 (1.14- 1.73)	0.0016
*(ref: no urine protein)*		

p value < 0.05 significant,

*Includes, homemakers (housewives), retired, unemployed and others.

**♦**Diabetes Mellitus (DM) = fasting blood sugar ≥ 126 or non-fasting blood sugar ≥ 200 or if self reported D.M or on ant diabetic medication.

Serum creatinine and waist circumference are taken as a continuous variable.

## Discussion

The main finding of this study is that hypertension prevalence in our SEEK cohort is 43.5%, and 41.5% of subjects had blood pressure in the range of “pre-hypertension”.

The major predictors of hypertension included age >40 years, male gender, BMI of ≥ 23 Kg/M^2^, waist circumference, sedentary occupation, proteinuria, and increased serum creatinine, proteinuria and CKD were observed in 19% and 23.5% of HTN subjects, respectively. Nearly two-thirds of the cohort with hypertension was overweight or obese compared to only approximately 40% of those without HTN. Collectively, these observations support the idea that hypertension is an important public health issue in developing countries and it has important associations with both kidney disease and obesity. In China, the largest and most populous country in Asia, HTN was identified as the major causes of death [20-22].

Both Asia and Africa are witnessing this increasing prevalence due to acculturation. Rapid and often uncontrolled urbanization leads to exposure to many health risks, including poor sanitation and environmental risk factors. However, it also leads to changes in dietary and lifestyle changes leading to decreased physical activity and increased risk of obesity and greater risk of hypertension. Most of the increase in the prevalence of HTN in the developing countries especially in the South East Asia has been attributed to the increase in obesity [23]. The increased prevalence of HTN in urban compared to the rural populations has been also shown in several other studies [24-26] including a recent meta-analysis [27]. It showed that the prevalence of HTN varied between 4.5 to 45% in rural populations, and a higher prevalence (13.9–48.2%) in the urban populations, with a significant increasing trend over time.

Excess body weight has been identified as an important independent risk factor for hypertension and related complications of CVD [28]. Several reports suggest that among the Chinese and South Asian population a higher prevalence of dyslipedemia, metabolic syndrome, type II diabetes mellitus and CVD are observed at a much lower value of BMI than the Europeans [28,29]. The normal range of BMI cutoff points derived in the western population may be misleading when used for these ethnic groups [29]. An Asian cut off for obesity and abdominal obesity among different ethnic populations was also defined by the International Diabetes Federation consensus group in 2004 [17]. This cut-off was much lower for the Chinese, Japanese, South Asian population as compared to the European population [30]. He et al. observed that waist circumference adds additional risk information to that of BMI in Chinese adults [31]. Obesity by conventional standards (17.9%) and Asian standards (33%) was prevalent among HTN subjects. However, 68.7% of the HTN population was overweight or obese compared to 42.9% of those without HTN. Further, we observed overweight or obese (BMI ≥ 23 Kg/M^2^) individuals to have 1.37 times higher prevalence of HTN compared to those with lean or normal BMI (< 23 Kg/M^2^). In the KEEP study, 50.8% of HTN subjects were obese using the conventional cut off [32] Also, waist circumference as a continuous variable revealed a significantly higher positive association with HTN. Given the propensity of Indian population to develop hypertension at a much lower body weight and waist circumference, urgent and concerted prevention policy development and implementation including educational intervention for improving diet and exercise is necessary.

Association of HTN with diabetes mellitus, proteinuria and CKD was high in our cohort. Kidney disease can be the cause or consequence of hypertension. Proteinuria is the earliest indication of kidney disease among diabetic subjects and has been observed in about 20- 40% of diabetic subjects without known kidney disease [33,34]. But prevalence of albuninuria among hypertensive individuals screened in large populations has been reported in 8–11.5% in the AusDIAB and Prevention of Renal and Vascular End Stage Disease (PREVEND) studies [35-37]. The relationship between hypertension and proteinuria has however not been explored in many epidemiologic studies reported from India [10,12,13]. Since these were screening camps, and the fact the renal involvement can be a cause or effect of hypertension, it is difficult to assess if the renal involvement is a predictor or a complication of HTN in our cohort. However, it is important to note this association was observed in a significant number of subjects in the cohort. With adequate management of both HTN and proteinuria, further complications of CVD, retinopathy as well as ESRD can be prevented. Training general practitioners to carry out a simple urine dip-stick test among hypertensive and diabetic subjects would help in detecting these cases early and preventing the progression.

Despite the design of the study wherein a bias may exist for more people with higher awareness of HTN to come to attend the screening camps, we observed a low awareness and control of HTN. Our study demonstrates a much lower HTN awareness than that reported from the USA by Burt et al., an awareness rate of 73% in the National Health and Nutrition Examination Surveys (NHANES III-1988 –1991, [38,39]. Low awareness of HTN may reflect disparities in health care access, lack of education, the effect of poverty, or a combination of these and other factors. Low awareness of HTN has been observed in other developing countries including China and Pakistan [40-42]. In part, this is because of the low level of literacy and education, but also seems to reflect low level of access to medical care. Indeed, in South Asian countries, awareness correlated with poor access to treatment and therefore to control of hypertension [42]. The challenge posed by low level of awareness must be overcome for prevention through strategies targeted at education and promotion. This lack of awareness underscores the importance of organizing an aggressive community based screening as well as health education campaign to improve awareness of morbidity and mortality due to HTN among the population. Greater commitment and availability of resources targeted at health education will likely to be needed.

Our findings indicate that prevalence of HTN was high in the screened population at 43.5%, with significant center to center variation in prevalence. This effect was observed independently of other risk factors for hypertension and could perhaps reflect regional differences in salt intake and/or other lifestyle practices. The fact that HTN increased with age and was higher for males was consistent with other reports [43,44]. In the meantime, the prevalence of hypertension of the SEEK study that we carried out in Thailand and Saudi Arabia were found to be 27.5% and 27.7% [45,46].

Our higher prevalence of HTN compared to some of the other reported series from India and other Asian countries like Pakistan and China may be related to the design of the study (camp module) as opposed to the domiciliary screening method used by others [4,13,42]. These differences in prevalence may also be related to the instrument used for the measurement of BP, the definition of hypertension used, and the genetic/anthropologic make up of the population screened. The findings also corroborate those by Keraney et al. where they reported that the “Global burden of Hypertension” will affect more than a quarter of the world’s adult population with HTN in 2000, and was projected to be increased by 60% by about 2025, the population burden being higher in the developing countries [4].

High prevalence of pre-hypertension (41.5%) in the screened population is also worrisome. A collaborative meta-analysis of individual participant data from one million adults with no previous vascular disease recorded at baseline in 61 prospective observational studies of blood pressure and mortality during 12.7 million person-years at risk was analyzed. The authors observed that throughout middle and old age, blood pressure is strongly and directly related to vascular (and overall) mortality, without any evidence of a threshold down to at least 115/75 mmHg [47]. The Trial Of Preventing Hypertension (TROPHY) also echoed the same observations that the CVD risk begins to rise even before the diagnosis of HTN is made [48]. The JNC 7 calls for routine blood pressure measurement at least once every 2 years for adults with pre-hypertension [8] and the American Heart Association issued similar recommendations for adults beginning at age 20 years for primary prevention of hypertension [49]. Surveillance of this pre-HTN population particularly among those >40 years of age for early detection of HTN will be essential.

This study had strengths and limitations. Its major strengths were the large sample size and the national distribution of health screening camps, although we did not have representation in the eastern parts of India. We believe that training of staff and use of standardized measurements also reduced the possibility of measurement bias. The main limitation was that we used a camp based methodology for screening rather than domiciliary screening and this may bias in favor of higher rates of disease. Nevertheless, our data adds information on risk factors, the burden of hypertension in the population. Further, this method has been used successfully as a cost effective method of detecting positive cases for screening large populations in the USA for the prevalence of CKD, hypertension and diabetes in the KEEP study [32]. An average of two measurements at a single visit is considered acceptable for an epidemiologic study using screening camps methodology and has been used by other surveys in the USA and other countries [28,50]. We defined HTN as EBP/self reported medications for HTN/self reported HTN as a positive answer to the question “have you ever been told that you have HTN”. This differs from the definition used by some of the previously reported studies that define HTN as EBP and/or self reported medications for HTN. A single spot urine sample and absence of quantification of albuminuria was also a limitation.

## Conclusions

In conclusion, HTN is an important public health problem in India. Reliable information about the prevalence of HTN is germane to the planning of any preventive or therapeutic strategies for HTN in a community. Low level of awareness and control in the HTN population makes it imperative to bring about a change in the way health related education should be brought to this population. An emphasis on education that is related to the control of modifiable predictors of hypertension, such as reducing obesity and abdominal obesity by diet and increasing physical exercise (especially for those involved in sedentary occupations) should be considered. Limiting salt consumption coupled with other lifestyle changes are likely to be one of the cost-effective population-based strategies and need to be considered as part of the public health agenda in India. If scarce resources are to be utilized optimally, control of HTN will help in improving the management of both CVD and CKD.

## Competing interests

The authors declare that they have no competing interests.

## Authors’ contributions

BVM and AKS – conceived and designed the study; AKS – acquired funding, supervised the overall execution of the study; BVM – cleaned the database; YMKF and SRKR – analyzed and interpreted the data: YMKF, BVM, and AKS – wrote the manuscript: All other co-authors – conducted and supervised the study procedures and the operational execution in the local Indian centers. All authors read and approved the final manuscript.

## Pre-publication history

The pre-publication history for this paper can be accessed here:

http://www.biomedcentral.com/1471-2369/15/42/prepub

## Supplementary Material

Additional file 1 SEEK Project – Screening Questionnaire. Click here for file

## Participating centers

Hinduja Hospital, Mumbai

Vivekananda Memorial Hospital, H.D.Kote, Saragur, Mysore

Manipal Institute of Nephrology and Urology, Bangalore

Deep Hospital, Ludhiana

Indraprastha Apollo Hospital, New Delhi

Bombay Hospital, Mumbai

Fortis Flt. Lt. Rajan Dhall Hospital, New Delhi

Bhopal Memorial Hospital & Research Center, Bhopal

PVS Memorial Hospital, Cochin

Institute of Medical Sciences, BHU, Varanasi

Muljibhai Patel Urological Hospital, Nadiad

Sir Ganga Ram Hospital, New Delhi

Wockhardt Hospitals, Bangalore

Kanpur Rotary Kidney Foundation, Kanpur

Lilavati Hospital, Mumbai

Dr. R.M.Lohia Hospital, New Delhi

Sanjay Gandhi Postgraduate Institute of Medical Sciences, Lucknow

Andhra Medical College and King George Hospital, Vishakhapatanam

## References

[B1] Murray CJ Lopez AD Mortality by cause for eight regions of the world: global burden of disease study Lancet 1997 349 9061 1269 1276 10.1016/S0140-6736(96)07493-4 9142060

[B2] Murray CJ Lauer JA Hutubessy RC Niessen L Tomijima N Rodgers A Lawes CM Evans DB Effectiveness and costs of interventions to lower systolic blood pressure and cholesterol: a global and regional analysis on reduction of cardiovascular-disease risk Lancet 2003 361 9359 717 725 10.1016/S0140-6736(03)12655-4 12620735

[B3] The sixth report of the joint national committee on prevention, detection, evaluation, and treatment of high blood pressure Arch Intern Med 1997 157 21 2413 2446 9385294 10.1001/archinte.157.21.2413

[B4] Kearney PM Whelton M Reynolds K Muntner P Whelton PK He J Global burden of hypertension: analysis of worldwide data Lancet 2005 365 9455 217 223 10.1016/S0140-6736(05)17741-1 15652604

[B5] Lopez AD Assessing the burden of mortality from cardiovascular diseases World Health Stat Q 1993 46 2 91 96 8303909

[B6] Ezzati M Lopez AD Rodgers A Vander Hoorn S Murray CJ Comparative Risk Assessment Collaborating Group Selected major risk factors and global and regional burden of disease Lancet 2002 360 9343 1347 1360 10.1016/S0140-6736(02)11403-6 12423980

[B7] Five-year findings of the hypertension detection and follow-up program. I. reduction in mortality of persons with high blood pressure, including mild hypertension. hypertension detection and follow-up program cooperative group JAMA 1997 277 2 157 166 8990344

[B8] Chobanian AV Bakris GL Black HR Cushman WC Green LA Izzo JL Jr Jones DW Materson BJ Oparil S Wright JT Jr Roccella EJ National Heart, Lung, and Blood Institute Joint National Committee on Prevention, Detection, Evaluation, and Treatment of High Blood Pressure The seventh report of the joint national committee on prevention, detection, evaluation, and treatment of high blood pressure: The JNC 7 report JAMA 2003 289 19 2560 2572 10.1001/jama.289.19.2560 12748199

[B9] Reddy KS Yusuf S Emerging epidemic of cardiovascular disease in developing countries Circulation 1998 97 6 596 601 10.1161/01.CIR.97.6.596 9494031

[B10] Malhotra P Kumari S Kumar R Jain S Sharma BK Prevalence and determinants of hypertension in an un-industrialised rural population of north india J Hum Hypertens 1999 13 7 467 472 10.1038/sj.jhh.1000864 10449211

[B11] Gupta R Sharma AK Prevalence of hypertension and subtypes in an Indian rural population: clinical and electrocardiographic correlates J Hum Hypertens 1994 8 11 823 829 7853325

[B12] Gupta R Guptha S Gupta VP Prakash H Prevalence and determinants of hypertension in the urban population of jaipur in western india J Hypertens 1995 13 10 1193 1200 10.1097/00004872-199510000-00014 8586811

[B13] Das SK Sanyal K Basu A Study of urban community survey in India: growing trend of high prevalence of hypertension in a developing country Int J Med Sci 2005 2 2 70 78 15968343 10.7150/ijms.2.70 PMC1145137

[B14] WHO Expert Consultation Appropriate body-mass index for asian populations and its implications for policy and intervention strategies Lancet 2004 363 9403 157 163 14726171 10.1016/S0140-6736(03)15268-3

[B15] Pickering TG Hall JE Appel LJ Falkner BE Graves JW Hill MN Jones DH Kurtz T Sheps SG Roccella EJ Council on High Blood Pressure Research Professional and Public Education Subcommittee, American Heart Association Recommendations for blood pressure measurement in humans: an AHA scientific statement from the council on high blood pressure research professional and public education subcommittee J Clin Hypertens (Greenwich) 2005 7 2 102 109 10.1111/j.1524-6175.2005.04377.x 15722655 PMC8109470

[B16] Levey AS Coresh J Greene T Marsh J Stevens LA Kusek JW Van Lente F Chronic Kidney Disease Epidemiology Collaboration Expressing the modification of diet in renal disease study equation for estimating glomerular filtration rate with standardized serum creatinine values Clin Chem 2007 53 4 766 772 10.1373/clinchem.2006.077180 17332152

[B17] Expert Committee on the Diagnosis and Classification of Diabetes Mellitus Report of the expert committee on the diagnosis and classification of diabetes mellitus Diabetes Care 2003 26 1 S5 S20 12502614 10.2337/diacare.26.2007.s5

[B18] Vassalotti JA Stevens LA Levey AS Testing for chronic kidney disease: a position statement from the national kidney foundation Am J Kidney Dis 2007 50 2 169 180 10.1053/j.ajkd.2007.06.013 17660017

[B19] Shtatland ES Barton MB An Information Gain Measure of Fit in PROC LOGISTIC 1998 1194 1199

[B20] He J Gu D Wu X Reynolds K Duan X Yao C Wang J Chen CS Chen J Wildman RP Klag MJ Whelton PK Major causes of death among men and women in china N Engl J Med 2005 353 11 1124 1134 10.1056/NEJMsa050467 16162883

[B21] He FJ MacGregor GA Effect of modest salt reduction on blood pressure: a meta-analysis of randomized trials. Implications for public health J Hum Hypertens 2002 16 11 761 770 10.1038/sj.jhh.1001459 12444537

[B22] Mittal BV Singh AK Hypertension in the developing world: challenges and opportunities Am J Kidney Dis 2010 55 3 590 598 Epub 2009 Dec 5. Review. PubMed PMID: 19962803 10.1053/j.ajkd.2009.06.044 19962803

[B23] Silventoinen K Sans S Tolonen H Monterde D Kuulasmaa K Kesteloot H Tuomilehto J WHO MONICA Project Trends in obesity and energy supply in the WHO MONICA project Int J Obes Relat Metab Disord 2004 28 5 710 718 10.1038/sj.ijo.0802614 15007395

[B24] Dutta A Ray MR Prevalence of hypertension and pre-hypertension in rural women: a report from the villages of West Bengal, a state in the eastern part of India Aust J Rural Health 2012 20 4 219 225 10.1111/j.1440-1584.2012.01287.x 22827431

[B25] Kaur M Blood pressure trends and hypertension among rural and urban Jat women of Haryana India Coll Antropol 2012 36 1 139 144 PubMed PMID: 22816211 10.5671/ca.2012361s.139 22816211

[B26] Momin MH Desai VK Kavishwar AB Study of socio-demographic factors affecting prevalence of hypertension among bank employees of Surat City Indian J Public Health 2012 56 1 44 48 10.4103/0019-557X.96970 22684172

[B27] Devi P Rao M Sigamani A Faruqui A Jose M Gupta R Kerkar P Jain RK Joshi R Chidambaram N Rao DS Thanikachalam S Iyengar SS Verghese K Mohan V Pais P Xavier D Prevalence, risk factors and awareness of hypertension in India: a systematic review J Hum Hypertens 2012 13 [Epub ahead of print] PMID: 22971751 10.1038/jhh.2012.33 22971751

[B28] Jafar TH Chaturvedi N Pappas G Prevalence of overweight and obesity and their association with hypertension and diabetes mellitus in an indo-asian population CMAJ 2006 175 9 1071 1077 10.1503/cmaj.060464 17060656 PMC1609152

[B29] Razak F Anand SS Shannon H Vuksan V Davis B Jacobs R Teo KK McQueen M Yusuf S Defining obesity cut points in a multiethnic population Circulation 2007 115 16 2111 2118 10.1161/CIRCULATIONAHA.106.635011 17420343

[B30] Alberti KG Zimmet P Shaw J IDF Epidemiology Task Force Consensus Group The metabolic syndrome--a new worldwide definition Lancet 2005 366 9491 1059 1062 10.1016/S0140-6736(05)67402-8 16182882

[B31] Wildman RP Gu D Reynolds K Duan X Wu X He J Are waist circumference and body mass index independently associated with cardiovascular disease risk in chinese adults? Am J Clin Nutr 2005 82 6 1195 1202 16332651 10.1093/ajcn/82.6.1195

[B32] Rao MV Qiu Y Wang C Bakris G Hypertension and CKD: kidney early evaluation program (KEEP) and national health and nutrition examination survey (NHANES), 1999–2004 Am J Kidney Dis 2008 51 4 Suppl 2 S30 S37 18359406 10.1053/j.ajkd.2007.12.012

[B33] Tapp RJ Shaw JE Zimmet PZ Balkau B Chadban SJ Tonkin AM Welborn TA Atkins RC Albuminuria is evident in the early stages of diabetes onset: results from the Australian diabetes, obesity, and lifestyle study (AusDiab) Am J Kidney Dis 2004 44 5 792 798 10.1053/j.ajkd.2004.07.006 15492944

[B34] Parving HH Lewis JB Ravid M Remuzzi G Hunsicker LG DEMAND investigators Prevalence and risk factors for microalbuminuria in a referred cohort of type II diabetic patients: a global perspective Kidney Int 2006 69 11 2057 2063 10.1038/sj.ki.5000377 16612330

[B35] Atkins RC Polkinghorne KR Briganti EM Shaw JE Zimmet PZ Chadban SJ Prevalence of albuminuria in Australia: the AusDiab kidney study Kidney Int Suppl 2004 92 92 S22 S24 15485411 10.1111/j.1523-1755.2004.09206.x

[B36] Hillege HL Janssen WM Bak AA Diercks GF Grobbee DE Crijns HJ Van Gilst WH De Zeeuw D De Jong PE Prevend Study Group Microalbuminuria is common, also in a nondiabetic, nonhypertensive population, and an independent indicator of cardiovascular risk factors and cardiovascular morbidity J Intern Med 2001 249 6 519 526 10.1046/j.1365-2796.2001.00833.x 11422658

[B37] de Zeeuw D Parving HH Henning RH Microalbuminuria as an early marker for cardiovascular disease J Am Soc Nephrol 2006 17 8 2100 2105 10.1681/ASN.2006050517 16825327

[B38] Burt VL Cutler JA Higgins M Horan MJ Labarthe D Whelton P Brown C Roccella EJ Trends in the prevalence, awareness, treatment, and control of hypertension in the adult US population. data from the health examination surveys, 1960 to 1991 Hypertension 1995 26 1 60 69 10.1161/01.HYP.26.1.60 7607734

[B39] Burt VL Whelton P Roccella EJ Brown C Cutler JA Higgins M Horan MJ Labarthe D Prevalence of hypertension in the US adult population. results from the third national health and nutrition examination survey, 1988–1991 Hypertension 1995 25 3 305 313 10.1161/01.HYP.25.3.305 7875754

[B40] Ibrahim MM Rizk H Appel LJ el Aroussy W Helmy S Sharaf Y Ashour Z Kandil H Roccella E Whelton PK Hypertension prevalence, awareness, treatment, and control in Egypt. Results from the egyptian national hypertension project (NHP). NHP investigative team Hypertension 1995 26 6.1 886 890 7490144 10.1161/01.hyp.26.6.886

[B41] Wang Z Wu Y Zhao L Li Y Yang J Zhou B Cooperative Research Group of the Study on Trends of Cardiovascular Diseases in China and Preventive Strategy for the 21st Century Trends in prevalence, awareness, treatment and control of hypertension in the middle-aged population of China, 1992–1998 Hypertens Res 2004 27 10 703 709 10.1291/hypres.27.703 15785004

[B42] Ahmad K Jafar TH Prevalence and determinants of blood pressure screening in pakistan J Hypertens 2005 23 11 1979 1984 10.1097/01.hjh.0000187258.86824.00 16208138

[B43] Gupta R Trends in hypertension epidemiology in India J Hum Hypertens 2004 18 2 73 78 10.1038/sj.jhh.1001633 14730320

[B44] Singh RB Suh IL Singh VP Chaithiraphan S Laothavorn P Sy RG Babilonia NA Rahman AR Sheikh S Tomlinson B Sarraf-Zadigan N Hypertension and stroke in Asia: Prevalence, control and strategies in developing countries for prevention J Hum Hypertens 2000 14 10–11 749 763 11095165 10.1038/sj.jhh.1001057

[B45] Alsuwaida AO Farag YM Al Sayyari AA Mousa D Alhejaili F Al-Harbi A Housawi A Mittal BV Singh AK Epidemiology of chronic kidney disease in the Kingdom of Saudi Arabia (SEEK-Saudi investigators) - a pilot study Saudi J Kidney Dis Transpl 2010 21 6 1066 1072 PubMed PMID: 21060175 21060175

[B46] Ingsathit A Thakkinstian A Chaiprasert A Sangthawan P Gojaseni P Kiattisunthorn K Ongaiyooth L Vanavanan S Sirivongs D Thirakhupt P Mittal B Singh AK Thai-SEEK Group Prevalence and risk factors of chronic kidney disease in the Thai adult population: Thai SEEK study Nephrol Dial Transplant 2010 25 5 1567 1575 Epub 2009 Dec 27. PubMed PMID: 20037182 10.1093/ndt/gfp669 20037182

[B47] Lewington S Clarke R Qizilbash N Peto R Collins R Prospective Studies Collaboration Age-specific relevance of usual blood pressure to vascular mortality: a meta-analysis of individual data for one million adults in 61 prospective studies Lancet 2002 360 9349 1903 1913 12493255 10.1016/s0140-6736(02)11911-8

[B48] Nesbitt SD Julius S Leonard D Egan BM Grozinski M TROPHY Study Investigators Is low-risk hypertension fact or fiction? cardiovascular risk profile in the TROPHY study Am J Hypertens 2005 18 7 980 985 10.1016/j.amjhyper.2005.01.021 16053996

[B49] Pearson TA Blair SN Daniels SR Eckel RH Fair JM Fortmann SP Franklin BA Goldstein LB Greenland P Grundy SM Hong Y Miller NH Lauer RM Ockene IS Sacco RL Sallis JF Jr Smith SC Jr Stone NJ Taubert KA AHA guidelines for primary prevention of cardiovascular disease and stroke: 2002 update: consensus panel guide to comprehensive risk reduction for adult patients without coronary or other atherosclerotic vascular diseases. American Heart Association Science advisory and coordinating committee Circulation 2002 106 3 388 391 10.1161/01.CIR.0000020190.45892.75 12119259

[B50] Hajjar I Kotchen TA Trends in prevalence, awareness, treatment, and control of hypertension in the United States, 1988–2000 JAMA 2003 290 2 199 206 10.1001/jama.290.2.199 12851274

